# A modified surgical technique for reconstruction of an acute acromioclavicular joint dislocation

**DOI:** 10.4103/0973-6042.59973

**Published:** 2009

**Authors:** Anthony Marchie, Arun Kumar, Melanio Catre

**Affiliations:** Department of Orthopedics, Toronto East General Hospital, Toronto, Ontario, Canada

**Keywords:** Acromioclavicular joint dislocation, suture anchors

## Abstract

We report a modified surgical technique for reconstruction of coracoclavicular and acromioclavicular ligaments after acute dislocation of acromioclavicular joint using suture anchors. We have repaired 3 consecutive type III acromioclavicular dislocations with good results. This technique is simple and safe and allows anatomical reconstruction of the ligaments in acute dislocations.

## INTRODUCTION

A number of different surgical techniques have been described to treat acute acromioclavicular joint dislocation. We describe a technique to treat this injury using suture anchors.

## SURGICAL TECHNIQUE

**Figure 1:**
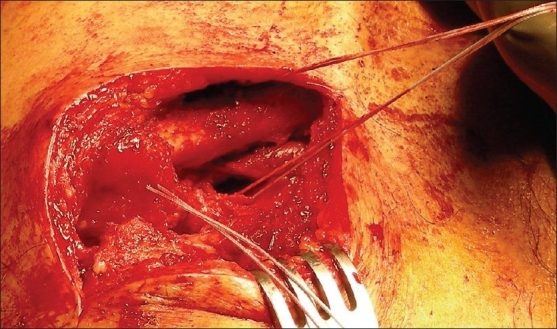
Per-operative photograph showing reconstruction of acromioclavicular and coracoclavicular ligaments using suture anchors

The patient is placed in a beach chair position under general anesthesia. A curvilinear incision is made over the lateral end of the clavicle and the acromion. The soft tissue dissection is carried down to the bone, raising full-thickness anterior and posterior flaps. The anterior fibers of the deltoid are usually stripped off the anterior surface of the lateral end of the clavicle following the injury. The acromioclavicular joint is exposed and cleared of any soft tissue interposition. At this stage the coracoid is identified and one corkscrew (5.5 mm in diameter, Arthrex) suture anchor is placed in the base of the coracoid. Now the coracoacromial ligament is identified and the medial part of the ligament is harvested and transferred to the lateral end of the clavicle using a corkscrew suture anchor in the lateral end of the clavicle followed by repair of acromioclavicular joint capsule, sometimes using a suture anchor. With acromioclavicular joint reduced, the sutures from the anchor in the coracoid are tied over the clavicle passing one suture anterior and another posterior to the clavicle [Figure 1]. The anterior portion of the deltoid is now repaired back to its origin.

The shoulder is immobilized in a shoulder immobilizer for a total period of 6 weeks. Passive mobilization and pendulum exercises of the shoulder are started at 3 weeks, followed by active, assisted exercises at 6 weeks. Patients return to full activities at 3 months, but contact sports are not allowed before 6 months.

## RESULTS

**Figure 2:**
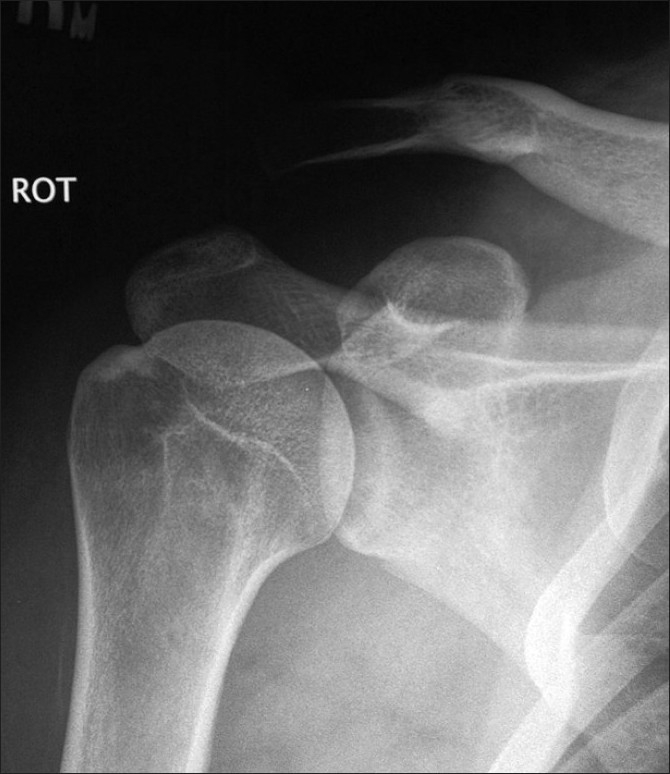
Radiograph of the shoulder showing type III dislocation of the acromioclavicular joint

**Figure 3:**
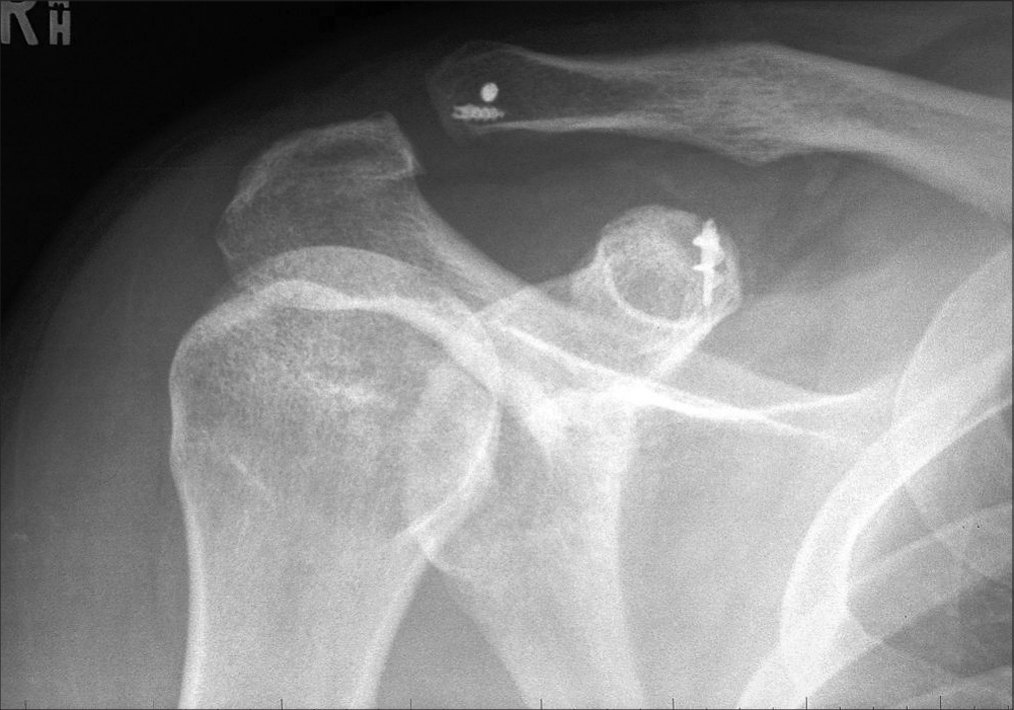
Radiograph of the shoulder 6 months after surgical stabilization using suture anchors, showing satisfactory reduction of the acromioclavicular joint

We have used this technique in 3 male patients with an average age of 27 years. All patients presented with an acute typeIII acromioclavicular dislocation following a fall [Figure 2]. All the 3 patients were physically very active. Two patients were involved in manual work requiring overhead lifting, and the third patient was a keen sportsman. Following surgery all patients achieved full range of movements and returned to routine daily activities at 3 months. Check radiograph at the follow-up at 6 months showed satisfactory reduction of the joint [Figure 3], and clinically all patients achieved pre-injury functional status.

## DISCUSSION

Various different methods have been used to surgically treat the complete dislocation (type III-VI) of the acromioclavicular joint, including threaded wires,[1] hook-plate fixation,[2] coracoclavicular screws,[3] coracoclavicular slings and loops,[4‐6] coracoacromial ligament graft with or without excision of the distal end of the clavicle[7,8] and more recently arthroscopic reconstruction of the joint.[9]

The use of metal work may be associated with fixation failure, implant breakage and need for removal of hardware.[2,3,10] To eliminate the problems of hardware, several authors have advocated the use of sutures or synthetic loops of absorbable or nonabsorbable material to stabilize the clavicle to the coracoid process.[4‐9] Reconstruction of the coracoclavicular ligament using sutures requires dissection around the coracoid process, risking injury to the neurovascular structures. To avoid the potential problems with the passage of sutures around the coracoid, Su *et al*. used suture anchors to reconstruct coracoclavicular ligament alone with good results in 11 patients. A cadaveric study has shown that similar stability can be achieved for coracoclavicular fixation with suture anchors or with sutures of similar material placed around the base of the coracoid for the treatment of acromioclavicular joint dislocation. However, potentially there is a decreased risk of neurovascular injury with the use of suture anchors as compared with passage of sutures around the coracoids.[11] Another cadaveric study has shown that suture anchors can provide strength similar to that of intact coracoclavicular ligament. In the same study, coracoacromial ligament transfer alone was found to be the weakest and it was recommended that this type of repair should be augmented with another form of coracoclavicular fixation.[12]

In our technique, by placing the suture anchor at the base of the coracoid, the line of pull of the sutures is as anatomical as with the native coracoclavicular ligaments. This also allows the remnant fibers of the torn coracoclavicular ligament to be more closely aligned and thus heal together in a more anatomical position. This technique allows anatomical reconstruction of both coracoclavicular and acromioclavicular ligaments and minimizes the risk of neurovascular injury. The use of medial half of the coracoacromial ligament further reinforces the repair. We have not treated chronic dislocations of acromioclavicular joint using suture anchors; but we have found this technique to be simple and safe, as well as a quick alternative mode to stabilize an acute dislocation of the acromioclavicular joint.
